# Supplementary material for: The Impact of Viral and Bacterial Co-Infections and Home Antibiotic Treatment in SARS-CoV-2 Hospitalized Patients at the Policlinico Tor Vergata Hospital, Rome, Italy
Source: Antibiotics (Basel). 2023 Aug 22;12(9):1348. doi: 10.3390/antibiotics12091348 (PMC10525365; doi:10.3390/antibiotics12091348)
Supplement: Supplementary file 1 [file antibiotics-12-01348-s001.zip › antibiotics-2555131-supplementary.pdf]

Table S1. Characteristics of HBsAg positive patients.

Pt	Age	Sex	Known	Previously treated	HBcAb	HBsAb	HBsAb quantitative (UI/ml)	HBeAg	HBeAb	HBsAg quantitative (UI/mL)	Viremia (UI/mL)	HDV serology	Treatment started	Outcome
1	43	F	NA	no	positive	positive	9.89	negative	positive	NA	<10	negative	no	LTFU
2	74	F	yes	no	positive	negative	0	negative	positive	2.30	<10	negative	entecavir	Follow-up
3	75	M	no	no	positive	negative	0.39	negative	positive	NA	NA	NA	no	Death
4	63	F	no	no	positive	negative	4.13	negative	positive	12.68	671	negative	entecavir	LTFU
5	69	M	no	no	positive	negative	0	negative	positive	31391.40	2380000	negative	tenofovir	Follow-up
6	68	M	no	no	positive	negative	0	negative	positive	NA	<10	negative	no	LTFU

Pt: patients ID number; F: female; LTFU: lost at follow-up; M: male; NA: not available; HBcAb: Aantibodies anti-hepatitis B core; HBsAb: antibodies anti-hepatitis S antigen; HBeAg: Hepatitis e antigen; HBeAb: antibodies anti-hepatitis e antigen; HBsAg: Hepatitis B surface antigen; HDV: Hepatitis D virus

Table S2. Characteristics of anti-HCV positive patients.

Pt	Age	Sex	Known	Previously treated	Viremia (IU/mL)	Outcome
1	56	F	no	no	18	LTFU
2	59	M	yes	no	negative	LTFU
3	50	M	no	no	51	LTFU
4	69	F	yes	yes - 2010	negative	No follow-up
5	78	F	yes	no	4330000	LTFU
6	48	F	no	no	negative	LTFU
7	33	M	no	no	4430	LTFU
8	60	M	no	no	negative	No follow-up
9	62	M	yes	yes - 2000	NA	LTFU
10	52	M	no	no	NA	LTFU

Pt: patients ID number; F: female; LTFU: lost at follow-up; M: male; NA: not available

Table S3. Characteristics of microbiological isolates from positive blood cultures.

Blood cultures considered as contaminant	21 → 10 *Staphylococcus hominis* (47.6%)<br>6 *Staphylococcus epidermidis* (28.6%)<br>2 *Staphylococcus capitis* (9.5%)<br>1 *Staphylococcus warneri* (4.8%)<br>1 *Corynebacterium acnes* (4.8%)<br>1 *Enterococcus faecalis* (4.8%)
Blood cultures treated as bloodstream infection	7 → 1 *Staphylococcus epidermidis*<br>1 *Staphylococcus aureus*<br>1 *Streptococcus suis*<br>1 *Haemophilus influenzae*<br>1 *Bacillus cereus*<br>1 *Bacillus amyloliquefaciens*<br>1 *Stenotrophomonas maltophila*
